# Target trial emulation on dexmedetomidine for critically ill patients: all that glitters is not gold

**DOI:** 10.62675/2965-2774.20250118

**Published:** 2025-05-04

**Authors:** Bruna Brandao Barreto, Lisa Burry, Dimitri Gusmao-Flores

**Affiliations:** 1 Hospital da Mulher Intensive Care Unit Salvador BA Brazil Intensive Care Unit, Hospital da Mulher - Salvador (BA), Brazil.; 2 Universidade Federal da Bahia Faculdade de Medicina da Bahia Salvador BA Brazil Postgraduate Program in Medicine and Health, Faculdade de Medicina da Bahia, Universidade Federal da Bahia - Salvador (BA), Brazil.; 3 Sinai Health Toronto Canada Sinai Health - Toronto, Canada.; 4 University of Toronto Leslie Dan Faculty of Pharmacy Toronto Canada Leslie Dan Faculty of Pharmacy, University of Toronto - Toronto, Canada.

Alpha-2 agonists have been used as anesthetic adjuncts for a very long time. Initially, drugs such as xylazine and detomidine were almost solely used for veterinary practice. Subsequent studies demonstrated the effectiveness of deep anesthesia in animals employing the newer, more potent, and selective alpha-2-agonists, such as the stereoisomer of medetomidine, dexmedetomidine (D-isomer).

In the 1990s, alpha-2 agonists became increasingly present in human anesthesiologists’ drug arsenal, with clonidine as the primary agent. Dexmedetomidine was soon included among the options for anesthesia and intensive care unit (ICU) use for postoperative patients (short-term use, i.e., < 24 hours). For mechanically ventilated patients who require prolonged sedation and agitation control, dexmedetomidine was still not considered an option until the early 2000s,^(1)^ with midazolam and propofol being considered preferred sedatives due to their more predictable awakening and titration. "The role of this new agent in sedation of ICU patients", according to guidelines at the time, remained "to be determined".

In 2007, in the context of growing concern about the role of sedation in the development of *delirium* and long-term brain dysfunction during and after critical illness, the MENDS trial was published, comparing dexmedetomidine with lorazepam.^(2)^ In this study, dexmedetomidine provided light sedation and more days free of delirium or coma. Since then, several randomized controlled trials have been conducted comparing dexmedetomidine to numerous sedatives and strategies (such as "usual care"). In a recent meta-analysis, 77 randomized controlled trials with dexmedetomidine were included, involving 11,997 critically ill patients, suggesting a reduced risk of *delirium* and agitation, and a reduced duration of mechanical ventilation.^(3)^ However, there was a greater risk of adverse events, such as hypotension and bradycardia.

Amidst all of these trials, in this issue of Critical Care Science, Serpa Neto et al.^(4)^ publish an target trial emulation (TTE) on dexmedetomidine to treat agitation in critically ill patients. This study employed an advanced methodology to replicate a randomized clinical trial using data initially gathered retrospectively. Although this approach is not novel, these study designs have become more prevalent over the past 5 years. Serpa Neto et al.^(4)^ examined the relationship between dexmedetomidine's early administration and the agitation resolution in patients admitted to ICUs. The study included all adult patients experiencing agitation who were consecutively admitted to ICUs from 2016 to 2021. Natural Language Processing was utilized to search for terms, words, or expressions in electronic medical records indicating agitation to identify eligible patients. Once the agitated patient population was defined, an intervention was identified, which was the administration of dexmedetomidine within 30 days of onset of agitation. Subsequently, two groups were analyzed using the TTE approach: 314 patients who received dexmedetomidine and 1,738 who did not. The primary outcome was the resolution of agitation within up to 30 days. The authors found that early dexmedetomidine use (i.e., within 12 hours) led to the resolution of agitation in approximately 93% of patients, compared to 72.1% in those who never received dexmedetomidine.

Although it seems reasonable and plausible for dexmedetomidine, a sedative drug, to reduce agitation like others in its class,^(5)^ these findings should be interpreted cautiously. Firstly, the sample size of patients using dexmedetomidine was small and even smaller -though unmentioned by the authors - was the group that began its use within 12 hours. The difference between these groups was marked in the case of patients who initiated use very early, rendering the result uncertain.

Secondly, while the results of this TTE appear to advocate for more extensive use of dexmedetomidine with broader inclusion criteria, we must not disregard recent evidence of increased mortality in individuals under 65 years old, as observed in the latest randomized controlled trial, SPICE III,^(6)^ and corroborated by the ADRESS trial,^(7)^ which included patients with refractory septic shock. These studies did not associate the increased mortality with the typical adverse events seen with this class of drug. Although the authors argue that, in this study, dexmedetomidine was not used as a sedative as it was in SPICE III trial, the mean doses in Serpa Neto's study^(4)^ (0.56mg/kg/h [0.34 - 0.87]) were higher than those used in the trial: 0.54mg/kg/hour [0.35 - 0.72] for patients younger than 65 years, and 0.44mg/kg/hour [0.27 - 0.64] for those older than 65years.

Thirdly, although TTE is a robust approach to designing and analyzing observational studies, it does not replace or dismiss the importance of conducting a well-designed clinical trial. Rather, TTE is an effective way to infer causality from observational data, especially in scenarios in which randomized experiments are not available due to being too expensive, infeasible, unethical, or untimely to support an urgent decision - none of which is the case for the use of dexmedetomidine. With that in mind, it is important that this study is evaluated in the context of the many randomized trials already published. Most importantly, we agree with the authors, the study should be considered hypothesis-generating until better evidence is provided.

While not necessarily a limitation of the study, the study's conclusion might lead to a potentially misleading reassurance: all causes of agitation should be treated with medication. The authors mention this issue, and we do agree with them on the reminder that, first and foremost, all instances of agitation should undergo a thorough evaluation to identify the root cause and potentially initiate a non-pharmacological intervention first. A strategy exploring non-pharmacological treatments is associated with improved and personalized bedside care and fewer adverse events and costs. However, in the case of persistent agitation, low-dose dexmedetomidine appears to be one alternative worth considering in the management of the agitated critically ill patient.
